# The Relationship Between Cultural Value Orientations and the Changes in Mobility During the Covid-19 Pandemic: A National-Level Analysis

**DOI:** 10.3389/fpsyg.2021.578190

**Published:** 2021-04-20

**Authors:** Selin Atalay, Gaye Solmazer

**Affiliations:** ^1^Department of Sociology, Izmir Bakircay University, Izmir, Turkey; ^2^Department of Psychology, Izmir Bakircay University, Izmir, Turkey

**Keywords:** COVID-19, social distancing, cultural value orientations, mobility changes, pandemic

## Abstract

This study investigated the relationship between cultural value orientations and country-specific changes in mobility during the Covid-19 pandemic. The aim was to understand how cultural values relate to mobility behavior during the initial stages of the pandemic. The aggregated data include Schwartz's cultural orientations, Gross Domestic Product (GDP) per capita, number of Covid-19 cases per million, and mobility change during the Covid-19 pandemic (Google Mobility Reports; percentage decrease in retail and recreation mobility, transit station mobility, workplace mobility and percentage mobility increase in residential areas). Regression analyses showed that, after controlling for economy and severity of disease, hierarchy was the primary factor reducing mobility, such as staying at home, and mobility in public spaces, such as avoiding retail and recreation sites (marginally significant). The results are discussed in the light of previous literature and the implications for social distancing measures.

## Introduction

The disease Covid-19, is caused by the severe acute respiratory syndrome coronavirus 2 (SARS-Cov-2) virus, the seventh virus from the Coronavirus family (Andersen et al., 2020). Coronaviruses caused the SARS epidemic in 2002–2003 and the MERS epidemic in 2012 (Wu et al., 2020). The first Covid-19 cases were identified in December 2019 in Wuhan, China after five patients were admitted to hospital between December 18 and 29, 2019, one of whom died (Rothan and Byrareddy, 2020). On December 31, 2019, a pneumonia case with an unidentifiable cause was reported to the Wuhan office of the World Health Organization (WHO). On January 30, WHO declared the outbreak a public health emergency and named it Covid-19 on February 11, 2020 (World Health Organization, n.d.).

The average incubation period for SARS-Cov-2 is 5.2 days, after which some infected individuals show symptoms (Rothan and Byrareddy, 2020) whereas others remain asymptomatic (Day, 2020; Nishiura et al., 2020). Because the latter are unaware of their status, they become sources of contagion unless measures are taken to limit their mobility. Asymptomatic virus transmission is therefore referred to as the “Achilles' heel of Covid-19 pandemic control” (Gandhi et al., 2020, p. 2,159).

Several non-pharmaceutical public health measures can be taken to slow the spread of a disease, such as quarantines, community containment, social isolation, and social distancing (Wilder-Smith and Freedman, 2020). These methods limit interaction between individuals to prevent contagion. Social distancing or physical distancing refers to measures taken to keep individuals apart by avoiding frequent physical contact and visiting crowded places (Centers for Disease Control and Prevention, n.d.). Relatedly, Smith and Branscum (2020) conceptualized social distancing behaviors under three categories (i.e., keeping physical distance with others, avoiding crowded places, and staying at home) in the context of Covid-19. Social distancing involves behavioral intervention strategies implemented by individuals themselves and by governments. The aim is to reduce contact between already infected and non-infected persons (Toxvaerd, 2020).

At the beginning of a pandemic, when a vaccine is unavailable and there is a limited supply of antiviral drugs, social distancing is a significant measure to prevent disease spread. By delaying the peak of the pandemic, social distancing protects the healthcare system from being overwhelmed, thereby enabling better care for patients until a vaccine or drug can be manufactured (Fong et al., 2020). Citizens can voluntarily use social distancing methods if they are informed about them. However, governments may also restrict their mobility to contain the pandemic (McGorty et al., 2007).

Social distancing actions that may be imposed by the government include closing workplaces, schools, places of worship, and places where crowds assemble (McGorty et al., 2007). Roads may be closed and travel restricted (Glass et al., 2006). Non-essential activity in places such as dining in restaurants, visiting entertainment venues, or gyms may be restrained. While the effectiveness of governmentally mandated social distancing methods requires the cooperation of individuals, voluntary social distancing is also significant in reducing human mobility during a pandemic (Courtemanche et al., 2020). Individuals may be encouraged to take responsibility, voluntarily refrain from social activity, and stay at home. Persons facing the risk of infection are shown to make behavioral changes by changing their contact patterns to avoid illness (Fenichel et al., 2011; Maloney and Taskin, 2020; Yan et al., 2020).

A report in March 2020 estimated that 3–4 months of moderate social distancing could save 1.7 million deaths from Covid-19 in the USA (Greenstone and Nigam, 2020). During the Covid-19 pandemic, studies show that governmental policies (Courtemanche et al., 2020; Siedner et al., 2020; Thu et al., 2020) and voluntary social distancing efforts are effective in containing the pandemic (Chudik et al., 2020). During the Covid-19 outbreak, governmental officials and public health authorities in different countries have employed various social distancing strategies. For example, countries like the Netherlands, Sweden, and the UK, have built their strategies more on trust, relying on their citizens to voluntarily restrict their mobility. Other countries have taken rigid measures. In Germany, for example, outdoor activities were allowed whereas Italy, Spain, and France imposed stricter social distancing. Some East Asian countries have imposed both strict measures and technological control (Organization for Economic Co-operation and Development, 2020). Nevertheless, to some extent, all measures rely on the public's compliance and responsible mobility behavior (Yan et al., 2020). Because the infectious disease is transmitted *via* human contact, restricting human mobility becomes a primary objective in public health policies (Fang et al., 2020). These policies have generally focused on decreasing mobility in public spaces while encouraging people to stay at home.

Aggregated mobility data collected by private companies is regarded as a significant source in understanding human mobility, for assessing the effectiveness of social distancing efforts and calibrating policies accordingly (Badr et al., 2020; Buckee et al., 2020). Due to the increase in the usage of smart phones, unlike the pandemics experienced in the past, it is relatively easier to quantify the changes in mobility behavior. Various studies on Covid-19 assess aggregated and anonymized mobility data collected by Apple (Cacciapaglia et al., 2020), Facebook (Thakkar et al., 2020), telecom operators (Badr et al., 2020), and by Google. Google mobility reports have already been used in studies analyzing mobility trends within countries (Basellini et al., 2020; Mellan et al., 2020; Vollmer et al., 2020) and for cross-country comparisons of mobility change. To illustrate, cross-country comparisons of the mobility data have been studied in relation to the number of cases and deaths (Yilmazkuday, 2020), different social distancing policies (Cacciapaglia et al., 2020), political trust (Bargain and Aminjonov, 2020), and economic outcomes (Alon et al., 2020).

In considering mobility change, country-specific factors, such as economic situation, the severity of the pandemic, and national culture, are all parameters affecting the general public's behavior. Regarding the voluntary and mandated distancing measures taken to regulate physical social interaction between individuals, this study focuses on culture as the primary factor influencing mobility behavior. Google mobility reports are used to quantify how individuals in different countries have reacted to the pandemic by changing their mobility behavior. Such behavior is a significant factor determining the course of the pandemic with important health consequences. Google mobility reports highlight the cross-national differences in mobility. Thus, cultural factors seem to be relevant to explain differences in mobility. Cultural factors may help deal with the everyday reality of this health threat, provide a meaningful explanation and ways of expression for this unexpected situation, and prevent group members from acting in ways that increase contagion and illness. In line with this argument, as Inman et al. (2017) note, it is important to understand cultural factors to ensure the effectiveness of measures for preventing risky health behavior.

There is no unified definition of culture (Unger and Schwartz, 2008) but numerous definitions that define it from various perspectives (Kroeber and Kluckhohn, 1963; Johnson, 2007). Nevertheless, culture is generally referred to as a system of values, beliefs, and symbols (Peacock, 1981) that translates into behavior and the creation of artifacts (Kroeber and Parsons, 1958). Schwartz (2006, p. 138) views culture as a “rich complex of meanings, beliefs, practices, symbols, norms, and values prevalent among people in a society.” The definition implies that the abovementioned concepts are cultural manifestations. He argues that it is impossible to directly observe culture whereas a culture can be analyzed *via* its manifestations (Schwartz, 2014). For him, each society's value emphasis is the central characteristic of a given culture and provides a significant subject of study (Schwartz, 2006).

Cultural values are defined as “shared conceptions of what is good and desirable in the culture, the cultural ideals,” they are the “vocabulary of socially approved goals used to motivate action, and to express and justify the solutions chosen” (Schwartz, 1999, 2011, p. 26, 139). Values define the categories of “dangerous vs. safe,” “abnormal vs. normal,” “moral vs. immoral.” Values are interrelated and form systems or hierarchies (Hofstede, 2001, p. 6). Cultural values have a significant role in the functioning of societies and their social institutions (Knafo et al., 2011). They are the standards that determine action (de Mooij, 2017), guide the way individuals, policymakers, and groups select, evaluate, and explain their conduct. Cultural value emphases are shared to the extent that social actors such as government leaders, select the socially accepted conduct and can justify their actions to other social actors who share these conceptions (Schwartz, 1999). Enacting a total lockdown, mandating various strict social distancing measures, or expecting voluntary behavioral changes may be relevant in this regard. These chosen ways of conduct have to be accepted and justified in terms of the cultural value emphasis in a given society.

Schwartz categorizes value dimensions as a priori constructs, formulated as Weberian ideal types. Ideal types are methodological tools or “artificial” categories that do not exist in reality but provide a basis for comparison (Weber, 2005, p. 56). For Schwartz (2011, p. 471), cultural value “orientations are normative responses; they prescribe how institutions should function and how people should behave in order to deal best with the key problems societies face.” While these value orientations are relatively stable, they may change when adaptation is required to new social or environmental conditions (Schwartz, 2006). Values operate on multiple layers. In order to explain nation-level behavioral responses, the appropriate level of analysis is cultural values (Kasser, 2011).

Taking a functionalist perspective, Schwartz proposes seven cultural value dimensions designed as bipolar ideal types depending on the answers to three fundamental questions that all societies must answer (Schwartz, 2007, 2014, p. 550): Where are the boundaries between the individual and the group? How will individuals coordinate to produce while managing interdependencies between individuals and preserving the social fabric? How will the management of the appropriation of natural and human resources take place? Seven cultural value orientations are formed in relation to these social issues. They are conceptualized in a circular structure as interdependent dimensions, depending on conflict or congruence among them. To be clear, cultural value orientations which are close to each other in this circle have congruent characteristics, while cultural value orientations which are remote from each other have opposing characteristics (Schwartz, 2006; Sagiv et al., 2011). Each culture is situated along these dimensions.

The dimensions of embeddedness and autonomy form the poles of a scale that answers the first question on the relation between the group and the individual. Embeddedness refers to cultures in which individuals are defined by the collectivity and whose individual identity is a continuation of this collective identity. In societies where embeddedness is a core value, it is important to maintain the status quo and the traditional social order (Schwartz, 1999, 2011). Embeddedness is related to “tradition, social order, family security, obedient, reciprocation of favors” (de Mooij, 2017, p. 449), national security, honoring elders, and protecting the public image. In societies where autonomy is a central value, individuality is valued, and people are encouraged to express themselves as active agents. Autonomy is further categorized by the intellectual dimension related to ideas and thoughts and affective dimension related to feelings and emotions. Affective autonomy refers to valuing positive affective experiences, such as pleasure and excitement. In groups where affective autonomy is valued, individuals are free to seek self-fulfillment through these affective experiences. Affective autonomy is related to enjoying a varied and exciting life and seeking pleasure. In societies where intellectual autonomy is a core value, individuals are encouraged to follow their own intellectual paths while traits like broadmindedness creativity and curiosity are valued (Schwartz, 1999, 2006, 2011).

The dimensions of egalitarianism and hierarchy form the poles that answer the second question. This question is related to the issue on how societies guarantee “responsible behavior that will preserve the social fabric.” Egalitarianism is the core value in societies where individuals recognize each other as equals, feel responsible toward each other, and voluntarily cooperate in this respect. It is related to notions such as “world of peace, freedom, responsible, and helpful.” In hierarchical societies, however, social coordination is based on ascribed roles and individuals act according to moral obligations. Social control is stricter when individuals accept the unequal and hierarchical distribution of power and resources (Schwartz, 1999, 2006, p. 26 and 31). Power and authority are “expected and accepted” (de Mooij, 2017). “Cultures high on egalitarianism emphasize such values as equality, social justice, honesty, and loyalty. Cultures high on hierarchy emphasize authority, social power, wealth, and humility” (Schwartz, 2007, p. 54).

The dimensions of harmony and mastery form opposite poles on the scale that answers the third question—the extent to which social actors can control and change their environment. Harmony cultures value harmonizing with and preserving the social and natural environment. Notions such as “world of beauty, unity with nature” are central. Mastery cultures encourage individuals and groups to master, control, and change their environment, and exploit natural resources to realize their ends. Values such as peace and environmental protection are emphasized in cultures high in harmony whereas ambition, competitiveness, choosing own goals, social recognition, and courage are valued in cultures high on mastery (Schwartz, 1999, 2014, p. 31; Schwartz and Melech, 2000).

Previous studies have used Schwartz's cultural orientation theory as a framework for investigating another public health problem, namely road safety. Gaygisiz (2010) found positive links between certain cultural value orientations (i.e., embeddedness, hierarchy, and mastery) and aggregated traffic fatality rates and a negative link between traffic fatality rates and intellectual autonomy and egalitarianism. Similarly, Solmazer et al. (2016) showed that traffic fatality rates are negatively associated with egalitarianism, harmony, and intellectual autonomy but positively associated with embeddedness and hierarchy. These studies suggest that egalitarianism and intellectual autonomy reduce public health problems whereas embeddedness and hierarchy worsen them. Mastery and harmony have inconsistent effects.

Consistently, there is also empirical evidence indicating the relationship between various health behaviors and cultural value orientation (e.g., Deschepper et al., 2008; Mackenbach, 2014; Gaygisiz et al., 2018). Specifically, Mackenbach (2014) shows that, in the framework of Schwartz's cultural orientation theory, embeddedness was negatively related to taking influenza vaccination, whereas intellectual autonomy, affective autonomy, and egalitarianism were positively related to taking influenza vaccination in elderly population. This study also shows that there are similar findings for breast cancer screening. To be precise, embeddedness and hierarchy were negatively related to breast cancer screening while intellectual autonomy (non-significant), affective autonomy, and egalitarianism were positively related to cancer screening. Mastery and harmony indicate insignificant effects.

Based on these arguments, the present study investigated the relationship between cultural orientations and mobility change which is seen as a behavioral response to social distancing measures during the Covid-19 pandemic. Given the conceptual framework and above findings, we expected that egalitarianism, which would be related to making responsible behavioral adjustments in order to protect self and others who are seen as equals and intellectual autonomy, to put barriers between the self and the group in order to prevent infection, would be positively related to mobility decrease in public space and increase in staying at home. On the contrary, we expected that the polar value dimensions of hierarchy and embeddedness to be negatively related to mobility decrease in public space and increase in staying at home. Additionally, given the framework and the uniqueness of pandemic as a health threat that requires measures to be taken for long periods of time, i.e., minimizing social activity and maximizing staying at home is required for days or months, we expected affective autonomy to have a different effect to that given in the previous literature. Since affective autonomy involves seeking pleasure and enjoying life, we expected it to be negatively related to mobility decrease in public space and increase in staying at home. Nonetheless, we had no expectations on the relationship between harmony and mastery dimensions and mobility change.

## Method

The current study included seven cultural value dimensions (i.e., harmony, embeddedness, hierarchy, mastery, affective autonomy, intellectual autonomy, and egalitarianism) from Schwartz's framework presenting data collected from school teachers and students in 75 countries (Schwartz, 2008) and also data for country-specific mobility change during the Covid-19 pandemic from Google's website (Google LLC, n.d.). Google mobility reports are designed to aid public health authorities in understanding changes in mobility trends during the pandemic and to see whether policies for staying at home, working from home, and avoiding public spaces have been successful. This is expected to provide insights for future policy making. The data is anonymized and the posted mobility files present charts that display how mobility trends change over several weeks for specific geographical areas (Aktay et al., 2020). This data is collected from location history of mobile devices and aggregated from users who have turned on their location history settings (Chan et al., 2020). Google posts reports for over 130 countries online at intervals of 2–6 days and does not publish a report on a location or category where statistically significant level of data is unavailable (Mobility Report CVS Documentation, n.d.).

The reports display how the number of visits and length of stay in different types of locations change in respect to the baseline (Bargain and Aminjonov, 2020). Google defined a period prior to the global spread of Covid-19 as baseline and calculated the percentage change by comparing mobility on a certain date and the mobility defined as the baseline measure. The baseline measure for each country refers to its median mobility score for the respective day between January 3 and February 6, 2020 (Community Mobility Reports Help, n.d.).

Mobility changes for each country in the Covid-19 pandemic are represented as percentage changes with respect to six major location categories. These are, grocery and pharmacy (such as grocery and drug stores), parks (such as “national parks, public beaches, marinas, dog parks, plazas, and public gardens”) workplaces, transit stations (“public transport hubs such as subway, bus, and train stations”), retail, and recreation (such as “restaurants, cafes, shopping centers, theme parks, museums, libraries, and movie theaters”). The reports also display mobility in residential areas which is regarded as the “stay-at-home measure” (Yilmazkuday, 2020, p. 5).

In public places (“retail, recreation, eateries; groceries, pharmacies; transit; and parks”), randomly selected four pair of visits^1^ in terms of category and location are considered and reported. In residential areas and workplaces, the “relative frequency, time and duration of visits” are calculated. For places of residence, the average amount of time spent at homes in terms of hours and for workplaces, the number of users who spend more than 1 h at places of work is calculated and reported (Aktay et al., 2020, p. 2–3).

The mobility data used in this study for mobility changes posted by Google are for April 26 and May 7, 2020. These dates were selected during what might be considered as the initial stages of the pandemic, when Covid-19 was declared a pandemic by WHO and had spread throughout most of the world. On April 26, there were a total of 2,832,750 cases and 205,326 deaths recorded worldwide while, there were a total of 3,714,816 cases and 263,501 deaths on May 7. Also, on April 25, the date prior to the first selected Google report, all countries that had available mobility change data and value orientation data had already reported at least one case (Covid-19-data, n.d.). In April, which may be considered the initial stage of the outbreak, even though Covid-19 had already been declared a pandemic and most countries suffered worldwide, the study tried to focus on countries for which the disease became a reality with the announcement of the first case.

Mobility scores for each country were calculated by taking the mean of the mobility data from April 26 and May 7, 2020. Gross Domestic Product (GDP) per capita for 2019, obtained from the International Momentary Fund's (IMF) website (International Monetary Fund, 2019) was used to indicate each country's economic situation. Finally, deaths per million and total cases per million for each country, pertaining to April 25, 2020 and May 6, 2020, which are the days prior to the dates for the mobility change analysis, were obtained online (Covid-19-data, n.d.). This data presents total cases and total deaths for each country's population. Deaths per million and total cases per million for these countries were calculated by taking the mean of data from April 25, 2020 and May 6, 2020. These figures were regarded as indicators of the severity of the pandemic in each country and were taken as factors that affected the way the public perceived the health threat and acted accordingly.

**Figure 1:**
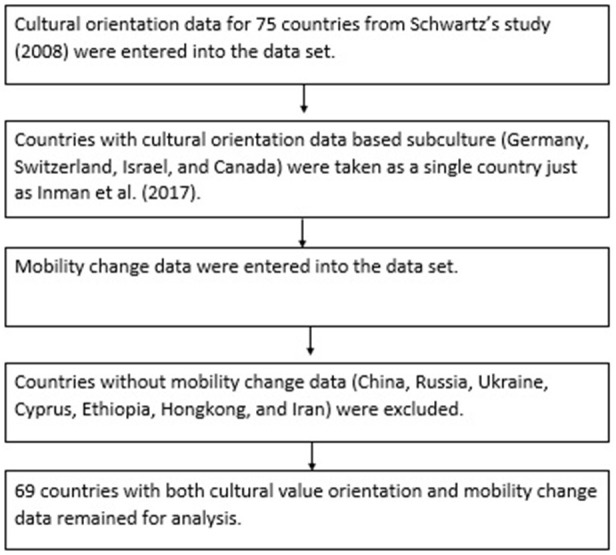
Data integration process.

Figure 1 depicts the data integration process after which 69 countries^2^ were available for analysis. Workplace, transit station, and retail, recreation, and residential area mobility changes were assessed in relation to cultural value orientations. All countries experienced a decrease in mobility except in Taiwan, where mean workplace mobility slightly increased. Thus, Taiwan was excluded from only the analyses pertaining to workplace mobility. All mobility change data (mean value of respective data for April 26 and May 6, 2020) were then re-formulated as percentage decrease or increase compared to the baseline measure. Only workplace, transit station, and retail and recreation mobility were examined as there was an overall decrease in mobility compared to the baseline. Residential area mobility was examined as a percentage increase in staying at home compared with the baseline measure.

### Data Analysis Strategy

Firstly, bivariate correlation analyses were conducted to assess the relationship between cultural value orientations and mobility, GDP, and disease severity (total cases per million and total deaths per million). Due to high correlation between total cases per million and total deaths per million (*r* = 0.83), only total cases per million was used as a measure of severity in the further analyses (partial correlation and regression analyses). Secondly, after controlling for GDP and total cases, partial correlation analyses were conducted to assess the stability of the observed associations. Before conducting the sequential regression analysis, multicollinearity was checked by VIF analysis. This indicated that embeddedness had a multicollinearity problem since its VIF value was >10 (Kutner et al., 2005; Paul, 2006). Hence, this variable was excluded from the sequential regression analyses, as suggested by Tabachnick and Fidell (2014). After excluding embeddedness, the VIF analysis showed that all VIF values for the remaining variables were lower than 5 (Paul, 2006), which indicates that there is no general problem. Finally, in the sequential regression, the total case variable was entered in the first step, GDP in the second step, and cultural orientations except for embeddedness in the third step.

## Results

### Descriptive Statistics

Table 1 shows the countries with the largest and smallest mobility changes. Mobility in retail and recreation and transit stations, i.e., public transportation, decreased in all cases. Workplace mobility in general decreased, apart from Taiwan, where it increased only on May 7 (shown in italics). Staying at home increased, except for Taiwan, where it decreased (shown in italics). While grocery and pharmacy shopping, and visits to parks generally decreased, this varied between countries. For grocery and pharmacy shopping, mobility increased in 65 countries (94.26%) and 58 countries (84.1%) for April 26 and May 7, respectively. For visits to parks, mobility decreased in 51 countries (73.9%) and 39 countries (56.5%) for April 26 and May 7, respectively. Our analyses focused on domains with decreasing mobility (i.e., retail and recreation, transit stations, workplace) as well as staying at home, which increased.

**Table 1:** Largest and smallest percentage increases and decreases for mobility.

	**Largest decrease**		**Smallest decrease**		**Largest increase**		**Smallest increase**	
	**April 26**	**May 7**	**April 26**	**May 7**	**April 26**	**May 7**	**April 26**	**May 7**
RRM	−95% (Peru, Serbia)	−84 (Peru)	−6% (South Korea)	−7% (South Korea)	–	–	–	–
GPM	−96% (Peru)	−65% (Bolivia)	−1% (Taiwan)	−2% (Australia, Egypt, Switzerland, Yemen)	73% (Poland)	34% (Chechia)	1% (Norway, South Korea)	1% (Brazil, Japan)
PM	−95% (Argentina)	−89% (Argentina)	−3% (Belgium)	−8% (Fiji)	82% (Sweden)	150% (Denmark)	4% (Taiwan)	2% (Bulgaria)
TSM	−93% (Peru)	**−79%** **(Jordan)**	−5% (South Korea)	−4% (South Korea)	–	–	–	–
WM	−74% (Peru)	**−83%** **(Singapore)**	−4% (Cameroon)	−1% (South Korea)	–	*7%* *(Taiwan*)	–	–
SH	–	*−1%* *(Taiwan)*	–	–	34% (Bolivia)	**48%** **(Singapore)**	3% (Chechia)	2% (South Korea)

*RRM, retail and recreation mobility; GPM, grocery and pharmacy mobility; PM, park mobility; TSM, transit stations mobility; WM, workplaces mobility; SH, staying at home. Exceptional cases are signified in bold*.

As the descriptive analysis showed that South American countries mostly had the largest decrease in public space mobility for April 26 and May 7. In contrast, public space mobility primarily increased in Northern Europe, where social distancing policies promoted voluntary personal measures, and East Asian countries near China, where the pandemic spread initially. Singapore is an exception here, although it is important to note that May 7 is a national holiday in Singapore and workplace restrictions were also introduced in Jordan on the same day, explaining why the country has the highest decrease in transit station mobility on that date (Holidays and Observances Around the World, n.d.).

Table 2 presents the correlations between the study variables. Among the cultural value orientations, egalitarianism which is related to valuing responsible and helpful behavior, cooperation and equality (Schwartz, 2006) was marginally significantly related to decrease in retail and recreation mobility; despite being insignificant, mastery which is related to social recognition, ambition, and competitiveness (Schwartz, 1999) showed a tendency toward being related to the decrease in transit station mobility and increase in staying at home. Harmony related to valuing peace and environmental protection and intellectual autonomy related to following own intellectual path, valuing creativity and curiosity (Schwartz, 2011) were negatively related to increase in staying at home, whereas hierarchy which is related to social power, authority, complying with obligations and embeddedness related to the maintenance of status quo and social order (Schwartz, 1999, 2011) were positively related to it, and despite being insignificant, affective autonomy showed a negative tendency toward being related to it. After controlling for GDP and total cases, despite being insignificant, hierarchy showed a tendency toward decrease in retail and recreation mobility and decrease in workplace mobility. Mastery was marginally significantly related to decrease in workplace mobility. Both hierarchy and mastery were positively related to decrease in transit station mobility. Embeddedness, hierarchy, and mastery were all positively related to increase in staying at home whereas harmony and intellectual autonomy were negatively related to it. Overall, hierarchy has the most powerful effect on mobility reduction, both generally and for staying at home specifically.

**Table 2:** Correlations among study variables.

	**1**	**2**	**3**	**4**	**5**	**6**	**7**	**8**	**9**	**10**	**11**	**12**	**13**
1. Harmony	1	−0.55^***^	−0.54^***^	−0.35^**^	0.22^+^	0.59^***^	0.28^*^	0.01	−0.12	−0.07	−0.37^*^		
2. Emb	−0.52^***^	1	0.38^**^	−0.16	−0.76^***^	−0.83^***^	−0.33^**^	−0.09	−0.01	0.01	0.27^*^		
3. Hierarchy	−0.55^***^	0.55^***^	1	0.35^**^	−0.19	−0.47^***^	−0.31^*^	0.21^+^	0.24^*^	0.22^+^	0.48^***^		
4. Mastery	−0.36^**^	−0.09	0.34^**^	1	0.27^*^	−0.04	−0.13	0.20	0.25^*^	0.24^++^	0.26^*^		
5. AA	0.26^*^	−0.85^***^	−0.40^**^	0.19	1	0.61^***^	0.02	0.10	0.11	0.08	−0.19		
6. IA	0.57^***^	−0.90^***^	−0.60^***^	−0.05	0.75^***^	1	0.25^*^	0.11	0.03	0.03	−0.31^*^		
7. Egalitarianism	0.35^**^	−0.57^***^	−0.50^***^	−0.15	0.33^**^	0.51^***^	1	0.11	0.04	−0.05	0.03		
8. DRRM	0.06	−0.06	0.10	0.12	0.02	0.09	0.23^++^	1	0.81^***^	0.85^***^	0.72^***^		
9. DTSM	−0.09	0.02	0.18	0.21^+^	0.03	0.01	0.10	0.83^***^	1	0.85^***^	0.80^***^		
10. DWM	0.02	−0.10	0.08	0.18	0.11	0.14	0.15	0.86^***^	0.84^***^	1	0.76^***^		
11. ISH	−0.32^**^	0.25^*^	0.39^**^	0.21^+^	−0.23^+^	−0.28^*^	0.07	0.76^***^	0.83^***^	0.76^***^	1		
12. Total cases	0.24^++^	−0.61^***^	−0.44^***^	−0.07	0.51^***^	0.58^**^	0.62^***^	0.21^+^	0.09	0.30^*^	0.05	1	
13. Total deaths	0.31^*^	−0.53^***^	−0.41^**^	−0.14	0.43^***^	0.55^***^	0.59^***^	0.21^+^	0.12	0.22^+^	0.02	0.83^***^	1
14. GDPpc	0.12	−0.63^***^	−0.38^**^	0.00	0.63^***^	0.56^***^	0.41^**^	−0.17	−0.14	−0.00	−0.21^+^	0.65^***^	0.43^***^

*Emb, embeddedness; AA, affective autonomy; IA, intellectual autonomy; DRRM, decrease in retail and recreation mobility (percentage decrease in retail and recreation mobility); DTSM, decrease in transit station mobility (percentage decrease in the transit station mobility); DWM, decrease in workplace mobility (percentage decrease in workplace mobility); ISH, increase in staying at home (percentage increase in staying at home). Total cases and total deaths data present total cases and total deaths per each country's population. The results presented on the right-hand side show the partial correlations among study variables after controlling for GDP and total cases; the results presented on the left-hand side show the bivariate correlations among study variables. ^++^p < 0.06 refers to marginally significant results in this study, while ^+^p < 0.10 refers to a tendency, even though the results are accepted as statistically insignificant*.

+ *p < 0.10*;

++ *p < 0.06*;

* *p < 0.05*;

** *p < 0.01*,

*** *p < 0.001*.

Regarding the relationship between cultural value orientations and total cases and deaths per million as indicators of the severity of Covid-19, harmony^3^, affective autonomy, intellectual autonomy, and egalitarianism were positively related to these measures whereas embeddedness and hierarchy were negatively related. Total cases per million was positively related to decrease in workplace mobility, which was also marginally significantly related to total deaths per million. That is, the higher the total number of cases and deaths, the less work mobility is in each country. Both total cases and total deaths were positively related to decrease in retail and recreation mobility.

### Regression Analyses

Four regression analyses were conducted to examine the relationships between cultural value orientations and changes in mobility during the Covid-19 pandemic after controlling for GDP and total cases per million as an indicator of the severity of Covid-19 in each country. The results are presented in Table 3.

**Table 3:** Model summary of sequential regression analysis examining relationships between cultural value orientations and mobility changes in the Covid-19 pandemic after controlling for total cases and GDP.

	**DRRM**		**DTSM**		**DWM**		**ISH**	
	**β**	***ΔR*^**2**^**	**β**	***ΔR*^**2**^**	**β**	***ΔR*^**2**^**	**β**	***ΔR*^**2**^**
Step 1		0.04^+^		0.01		0.09^*^		0.00
Cases	0.21^+^		0.09		0.30^*^		0.05	
Step 2		0.16^**^		0.07^*^		0.07^*^		0.11^**^
GDP	−0.53^**^		−0.34^*^		−0.35^*^		−0.43^**^	
Step 3		0.10		0.11		0.08		0.26^**^
Harmony	0.04		−0.08		0.04		−0.14	
Hierarchy	0.30^++^		0.27		0.25		0.38^*^	
Mastery	0.10		0.13		0.16		0.11	
AA	0.04		0.10		−0.01		−0.07	
IA	0.16		0.12		0.15		−0.11	
EGA	0.24		0.19		0.00		0.30^*^	

*DRRM, decrease in retail and recreation mobility (percentage decrease in the retail and recreation mobility); DTSM, decrease in transit station mobility (percentage decrease in the transit station mobility); DWM, decrease in workplace mobility (percentage decrease in the workplace mobility); ISH, increase in staying at home (percentage increase in staying at home); AA, affective autonomy; IA, intellectual autonomy; EGA, egalitarianism*.

+ *p < 0.10*;

* *p < 0.05*;

** *p < 0.01*.

*Embeddedness was excluded from the analyses due to multicollinearity. Total cases data present total cases per each country's population*.

++ *p < 0.06 refers to marginally significant results in this study*,

*while ^+^p < 0.10 refers to a tendency, even though the results are accepted as statistically insignificant*.

For retail and recreation mobility, model 1 was not significant^4^. Despite being insignificant, total cases showed a tendency toward being related to the decrease in retail and recreation mobility (β = 0.21, *p* = 0.088). Model 2 which included GDP per capita, added significant incremental variance in explaining decreases in retail and recreation mobility, Δ*R*^2^ = 0.16, *F*_change_ (1, 66) = 13.16, *p* = 0.001. GDP was significantly negatively related to decreases in retail and recreation mobility (β = −0.53, *p* = 0.001). Model 3, which included six cultural orientations, made no significant contribution to the equation. Only hierarchy was marginally significantly positively related to decreases in retail and recreation mobility (β = 0.30, *p* = 0.059).

For transit station mobility, model 1 was not significant. Total cases were not significantly related to decreases in transit station mobility. Model 2 which included GDP, added significant incremental variance in explaining decreases in transit station mobility, Δ*R*^2^ = 0.07, *F*_change_ (1, 66) = 4.79, *p* = 0.032. GDP was negatively related to decreases in transit station mobility (β = −0.34, *p* = 0.032). Model 3 made no significant contribution to the equation. That is, none of the cultural variables were related to the decrease in transit station mobility.

For workplace mobility, model 1 was significant, explaining 9% of the variance, *F*_(1, 66)_ = 6.59, *p* = 0.013). Total cases per million was positively related to the decreases in workplace mobility (β = 0.30, *p* = 0.013). That is, as the number of total cases increases, decrease in workplace mobility also increases. Model 2, which included GDP per capita, added significant incremental variance in explaining decline in workplace mobility [Δ*R*^2^ = 0.07, *F*_change(1, 65)_ = 5.46, *p* = 0.023]. GDP per capita was negatively related to the decrease in workplace mobility (β = −0.35, *p* = 0.023). Model 3 made no significant contribution to the equation. None of the cultural variables were positively related to the decline in workplace mobility.

For staying at home, model 1 was not significant. Total cases per million in this model was not significantly related to the increases in staying at home. Model 2 which included GDP per capita, added significant incremental variance in explaining the increase in staying at home, Δ*R*^2^ = 0.11, *F*_change_ (1, 66) = 7.89, *p* = 0.007. GDP per capita was negatively related to the increases in staying at home (β = −0.43, *p* = 0.007). Model 3 added significant incremental variance in explaining the increases in staying at home, Δ*R*^2^ = 0.26, *F*_change(6, 60)_ = 4.16, *p* = 0.001. Finally, in model 3, hierarchy (β = 0.38, *p* = 0.013) and egalitarianism (β = 0.30, *p* = 0.039) were positively related to increases in staying at home.

## Discussion

The aim of the present study was to investigate the relationships between Schwartz's cultural value orientations and mobility change during the Covid-19 pandemic as a measure of social distancing behavior. Mobility change was investigated under four categories (decrease in workplace mobility, decrease in transit station mobility, decrease in retail and recreation mobility, and increase in staying at home).

Descriptive statistics for the pandemic indicate cross-country differences in its severity. The present study investigated whether there is a cultural influence on behavioral responses to Covid-19 pandemic beyond these statistics. Not surprisingly, total cases per million, as an indicator of the severity of the disease in each country, was statistically significantly related to decrease in workplace mobility and showed a tendency toward being related to the decrease in retail and recreation mobility, despite being insignificant. This may be because, as the number of people diagnosed with Covid-19 increases, countries take more precautions, such as closing workplaces and cafeterias, to reduce mobility while certain services waver due to decreased customer demand. Interestingly, the statistics measuring the country specific severity of Covid-19 was generally unrelated to mobility changes, except for abovementioned effects.

As an indicator of each country's economic situation, GDP per capita was related to mobility change. Intuitively, as the stronger a country's economic situation is, the more it can transfer resources to interventions in the Covid-19 pandemic, such as for strategies to reduce mobility in the public space. Surprisingly, however, the effect of GDP was in the opposite direction to that predicted. Specifically, we found that GDP per capita was negatively related to all types of mobility reduction. This finding contradicts previous studies on various public health problems (e.g., Özkan and Lajunen, 2007; Solmazer et al., 2016), which show that the economy has a strong beneficial effect on public health problems. The present study documented a negative relationship between hierarchy and GDP per capita. The argument that the negative correlation between GDP per capita and mobility change may be partially interpreted as an effect of hierarchy was tested in the additional regression analyses^5^ in which cultural value orientations were entered in the first step, the number of total cases was entered in the second step, and GDP was entered in the final step.

The results showed that GDP has a unique effect on human mobility behaviors (see Appendix A). It is important to note that, despite being insignificant, we found only a tendency for negative relationship between GDP per capita and increase in staying at home implying that in the countries with higher GDP per capita, individuals did not stay at home as much as individuals in countries with a lower GDP per capita. The effect of GDP per capita on staying at home was strengthened in the regression analyses, implying that there is a suppression effect (Tabachnick and Fidell, 2014). Hence, this result should be interpreted cautiously. In addition, as discussed by Özkan and Lajunen (2011), economic situation may affect various variables including car ownership, quality of public transportation, open (green) space quality, population both directly and indirectly. These variables may explain the unexpected effect of GDP on human mobility behaviors. To illustrate, the country's with stronger economies may have larger open green space that enables different social functioning; thus, more mobility behaviors may be observed. This finding may be relevant to the initial disease outbreak and can be explored by further studies on the relationship between economy and mobility during the pandemic.

Surprisingly, the results suggest that hierarchy is the most important cultural value encouraging adaptive responses to the pandemic, such as for staying at home and avoiding public spaces. Specifically, hierarchy was marginally significantly and positively related to mobility reduction in retail and recreation after controlling for the economy and severity of disease. It was also positively related to increased staying at home. This indicates that, faced with health threat like a pandemic, culture can impose “socially responsible behavior” (Schwartz and Melech, 2000, p. 236). In the present study, mobility behavior during an unexpected and unprecedented Covid-19 pandemic (Lee et al., 2020) may be motivated by the actions of passively compliant individuals compelled to follow the rules imposed by respected authorities or they may be more inclined to alter their behavior according to the suggestions of authorities.

According to Kagitçibaşi and Cemalciler (2018), there may be a preference following natural disasters for an autocratic rather than democratic leader. Cohen et al. (2004) found that mortality salience leads individuals to assess a relationship-oriented leadership candidate who embodies egalitarianism more negatively and prefer charismatic and task-oriented leadership^6^ rather than relationship-oriented leadership. Consistent with their model, Jost et al. (2003, p. 366) assert that “several specific motives relating to the management of fear and uncertainty are associated with the ideology of political conservatism.” Jost et al. (2007) found that both uncertainty and threat increased political conservatism. Jost et al. (2003) argued that political conservation has two main dimensions, namely reluctance to change and approval of inequality. Since societies that value hierarchy emphasize authority and acceptance of inequality (Schwartz, 2007), these arguments seem to be relevant for hierarchy. Thus, it seems plausible that the Covid-19 pandemic creates uncertain conditions that make mortality salient; hence, uncertainty and the threat of dying may make a hierarchy cultural orientation more adaptive in responding to the pandemic.

Another possible explanation for this interesting finding concerns worry, defined as “a distributing cognition that a state of an object (macro or micro) in some domain of life (health, safety, etc.) will become (become more, or remain) discrepant from its desired state” (Schwartz et al., 2000, p. 311). Generally, results confirm that people in countries that value more hierarchy and less egalitarianism worry more about the self and in-groups. This is referred to as micro worry (e.g. “someone close to me being infected with AIDS”). On the other hand, people in countries with high egalitarianism worry more about their society and the world in general. This is called macro worry (e.g. “outbreak of a nuclear war”). Overall, egalitarianism is related to less micro worry but more macro worry while the reverse is true for hierarchy (Schwartz and Melech, 2000, p. 222). Extending this finding to the Covid-19 pandemic, it seems plausible that countries valuing hierarchy have more micro worries, such as someone close to me being infected with the Covid-19, whereas countries valuing egalitarianism have more macro worries like the outbreak of Covid-19. Just as these worries have different cultural origins, they may affect different outcome variables, such as mobility reduction. Specifically, the micro worries of people in societies that value hierarchy may encourage them to behave more adaptively to the pandemic.

This surprising result contradicts a previous study on road safety, which indicated that hierarchy decreases safety (Gaygisiz, 2010; Solmazer et al., 2016). Gaygisiz (2010) suggested that people in hierarchical societies may be less compliant with traffic regulations and rules since they think that these do not apply equally to everyone due to social hierarchy. Her results also showed that the detrimental effect of hierarchy was strengthened by lower governance quality. It thus seems plausible that people in societies characterized by hierarchy respect regulations, rules, and suggestions from the authorities related to the Covid-19 pandemic more since they regard them as applicable to everyone, along with strong enforcements, which are valid for everyone.

As predicted, we found that egalitarianism was positively related to increased staying at home in the regression. However, the bivariate and partial correlations between egalitarianism and increased staying at home after controlling for GDP and total cases per million suggest that there is no relationship between them. Rather, adding another cultural orientation to the equation enhances the importance of egalitarianism by reducing irrelevant variance in egalitarianism, meaning that there is a suppression effect (Tabachnick and Fidell, 2014). Hence, this result should be interpreted cautiously. Egalitarianism, the opposite ideal type to hierarchy—which in this study found to be the primary factor affecting mobility—is also a cultural value related to interdependencies between individuals. Even though egalitarianism, which is associated with an active interest in the welfare of all people, with an emphasis on equality (Schwartz, 2006), was expected to have a positive relationship with adaptive behavioral response to the Covid-19 pandemic, the findings suggest it is not a completely functional adaptive response. This may be because this pandemic is a unique public health emergency, unexpected by the public. The governmental response in societies that value hierarchy may have tended toward imposing strict measures followed by public compliance whereas governmental policies in societies that value egalitarianism may not have communicated the appropriate message to prompt compliance and responsible voluntary behavior. Countries need to employ culture bound social distancing measures. Thus, in egalitarian cultures, it is important to stress the importance of protecting both oneself and other people while prioritizing personal responsibility and caring for others as well as equality and social justice (Schwartz, 2006).

The findings have several implications. In general, the study revealed that countries' cultural value orientations have influenced mobility reduction during the Covid-19 pandemic. As Gaygisiz et al. (2018) suggest regarding antibiotics use, policy makers may use such findings to create more effective public health strategies for behavioral change and interventions for mobility reduction. There are some limitations in this study that need to be considered. The most important limitation concerns Google mobility data. This data is only collected from smart phone owners^7^ who have turned on their Google location history. The location accuracy may also vary between regions and for urban and rural places. The second limitation is that the relationship between societal value orientation and mobility reduction was tested at a national level. This could lead to the ecological fallacy, defined as “the confusion between within-system and between-system correlations” (Hofstede, 2001, p. 16). The third limitation is that although the selected days were while the disease was spreading actively to affect most of the world and that at least one case is reported by all countries under analysis, there are big differences between countries in terms of the severity of the disease. At this point, it is important to keep in mind that the present study used the severity of the disease as a control variable and reported the effects of cultural values after controlling for this variable. Despite this, there may be an interaction effect between cultural value orientations and severity of the pandemic such that cultural value orientations are associated with change in mobility when the severity of the pandemic is high but not when the severity is low. Hence, our analyses may not reveal cultural differences across countries where the severity of the pandemic is relatively low. Future studies could test this interaction effect between cultural value orientations and severity of the pandemic. The fourth limitation is that the mobility data is limited to 2 days. The final limitation is that these findings represent a short-term response to the pandemic. Different cultural values may be more effective in dealing with the pandemic in the long term.

Despite these limitations, we believe that this study contributes to the literature by showing the effects of cultural value orientations on social distancing behavior in the initial stages of the Covid-19 pandemic. A study investigating the relationship between Hofstede's cultural dimensions and cross-country changes in mobility on March 29, 2020 was published very recently (Huynh, 2020). However, as far as we are aware, our study is the first to analyze the relationship between Schwartz's cultural orientations and mobility during this pandemic, thereby providing a basis for understanding motivation in staying at home. Our findings may thus be taken into consideration when designing country specific social distancing measures.

## Data Availability Statement

The datasets presented in this study can be found in online repositories. The names of the repository/repositories and accession number(s) can be found in the article/supplementary material.

## Author Contributions

SA and GS: manuscript writing, literature review, data integration, and data analysis. All authors contributed to the article and approved the submitted version.

## Conflict of Interest

The authors declare that the research was conducted in the absence of any commercial or financial relationships that could be construed as a potential conflict of interest.

## Footnotes

^1^This does not affect accuracy since for example in the USA 99% of reported users make three or fewer visits daily (Aktay et al., 2020).

^2^Argentina, Austria, Australia, Belgium, Bolivia, Bosnia and Herzegovina, Brazil, Bulgaria, Cameroon, Canada, Chile, Colombia, Costa Rica, Croatia, Czechia, Denmark, Egypt, Estonia, Fiji, Finland, France, Georgia, Germany, Ghana, Greece, Hungary, Israel, India, Indonesia, Ireland, Italy, Japan, Jordan, Korea South, Latvia, Macedonia, Malaysia, Mexico, Namibia, Nepal, Netherlands, New Zealand, Nigeria, Norway, Oman, Pakistan, Peru, Philippines, Poland, Portugal, Romania, Senegal, Serbia, Singapore, Slovakia, Slovenia, South Africa, Spain, Sweden, Switzerland, Taiwan, Thailand, Turkey, Uganda, UK, USA, Venezuela, Yemen, and Zimbabwe.

^3^Harmony was marginally significantly related to total cases (*p* = 0.052).

^4^Model 1 approached conventional levels of statistical significance, *F*_(1, 67)_ = 2.99, *p* = 0.088.

^5^Four additional regression analyses were conducted to examine the relationships between GDP and changes in mobility during the Covid-19 pandemic after controlling for total cases per million and cultural value orientations to explore the unique effects of GDP and total cases on social distancing. The results are presented in Appendix A. The results indicated that total cases influenced workplace mobility after controlling for cultural value orientations. In addition, GDP influenced social distancing independently from total cases and the cultural value orientations.

^6^At this point, it is important to note that there is a preference for task-oriented leaders in both the control and mortality salience conditions.

^7^See Maloney and Taskin (2020, p. 17–18) who consider this limitation for using Google mobility data and provide a table for smartphone coverage in 50 countries.

## Appendix A

**Table A1:** Model summary of sequential regression analysis examining relationships between GDP and mobility changes in the Covid-19 pandemic after controlling for cultural value orientations and total cases.

	**DRRM**		**DTSM**		**DWM**		**ISH**	
	**β**	***ΔR*^2^**	**β**	***ΔR*^2^**	**β**	***ΔR*^2^**	**β**	***ΔR*^2^**
**Step 1**		0.14		0.11		0.10		0.3^**^
Harmony	0.12		–0.01		0.04		–0.12	
Hierarchy	0.32^+^		0.29		0.25		0.38^*^	
Mastery	0.14		0.16		0.17		0.12	
AA	–0.14		–0.04		–0.07		–0.15	
IA	0.16		0.10		0.24		–0.07	
EGA	0.33^*^		0.23		0.18		0.41^**^	
**Step 2**		0.02		0.00		0.08^*^		0.03
Cases	0.21		0.09		0.41^*^		0.25	
**Step 3**		0.13^**^		0.07^*^		0.06^*^		0.04^*^
GDP	–0.56^**^		–0.41^*^		–0.36^*^		–0.32^*^	

*DRRM, decrease in retail and recreation mobility (percentage decrease in retail and recreation mobility); DTSM, decrease in transit station mobility (percentage decrease in the transit station mobility); DWM, decrease in workplace mobility (percentage decrease in the workplace mobility); ISH, increase in staying at home (percentage increase in staying at home); AA, affective autonomy; IA, intellectual autonomy; EGA, egalitarianism*.

+ *p < 0.10*;

* *p < 0.05*;

** *p < 0.01*.

*Embeddedness was excluded from the analyses due to multicollinearity. Total cases data present total cases per each country's population. ^+^p < 0.10 refers to a tendency, even though the results are accepted as statistically insignificant*.
